# Explaining the Decrease of In-Hospital Mortality from Ischemic Stroke

**DOI:** 10.1371/journal.pone.0131473

**Published:** 2015-07-08

**Authors:** Jens Minnerup, Heike Wersching, Michael Unrath, Klaus Berger

**Affiliations:** 1 Department of Neurology, University of Münster, Albert-Schweitzer-Campus 1, 48149, Münster, Germany; 2 Institute of Epidemiology and Social Medicine, University of Münster, Albert-Schweitzer-Campus 1, 48149, Münster, Germany; National University of Singapore, SINGAPORE

## Abstract

**Background:**

Mortality from ischemic stroke has declined over time. However, little is known about the reasons for the decreased mortality. We therefore aimed to evaluate trends in in-hospital mortality and to identify factors associated with these trends.

**Methods:**

This study was based on a prospective database of 26 hospitals of the Stroke Register of Northwestern Germany, which included 73,614 patients admitted between 2000 and 2011. Time trends in observed (crude) and risk-adjusted in-hospital mortality were assessed. Independent factors associated with death after stroke were evaluated using multivariable logistic regression analysis.

**Results:**

The observed in-hospital mortality decreased from 6.6% in 2000 to 4.6% in 2008 (P < 0.001 for trend) and then remained fairly stable. The risk-adjusted mortality decreased from 2.85% in 2000 to 1.86% in 2008 (P < 0.01 for trend) and then increased to 2.32% in 2011. Use of in-hospital treatments including antiplatelets within 48 hours, antihypertensive therapy, statins, antidiabetics, physiotherapy and anticoagulants increased over time and was significantly associated with a decrease in mortality. The association of the year of admission with mortality became insignificant after adjustment for antiplatelet therapy within 48 hours (from OR 0.96; 95% CI, 0.94-0.98, to OR 0.99; 95% CI, 0.97-1.01) and physiotherapy (from OR 0.96; 95% CI, 0.94-0.97, to OR 0.99; 95% CI, 0.97-1.00).

**Conclusions:**

In-hospital mortality decreased by approximately one third between 2000 and 2008. This decline was paralleled by improvements in different in-hospital managements, and we demonstrated that it was partly mediated by early antiplatelet therapy and physiotherapy use.

## Introduction

Stroke is the second leading cause of death worldwide,[1] despite reports of a decrease in its case fatality over time.[2,3] Mortality trends can be affected by different factors, such as changes in patient characteristics, improved treatments, and advances in secondary prevention. Indeed, the decline in stroke mortality was particularly notable in the last decade,[3] the time period in which new stroke treatments have been established and were increasingly used thereafter. Proven therapies are particularly available for the acute phase after stroke, including thrombolysis with rtPA, antiplatelet agents within 48 hours, and stroke unit care.[4] But due to the demographic change in many countries the age distribution, disease severity, and the comorbidity profile of stroke patients have also significantly changed.[5] Thus, factors that explain the decline in stroke mortality are widely unclear.

The aims of the present study were to evaluate trends in in-hospital mortality over 11 years and to analyze patient and treatment related factors explaining these trends in a large German stroke register.

## Methods

Data were prospectively assessed within the Stroke Register of Northwestern Germany. Characteristics of this register have been described previously.[6] This registration is part of a legal act implemented in Germany through which hospitals participate in programs for quality assurance of acute clinical stroke care. The registry is not population-based and participation is open to all hospitals that treat patients with acute stroke. Although registration is optional, for reasons of quality assurance, hospitals with a stroke unit need to prove participation in a registry to be certified by the German Stroke Society. The register was started in 2000 and has rapidly grown since then. It includes urban and rural regions as well as departments of neurology and internal medicine from community and also academic hospitals. From the currently participating 155 hospitals we included those 26 hospitals that documented their stroke patients since the year 2000 and in at least 8 years during the study period between January 2000 and December 2011 (S1 Appendix). All ischemic stroke patients admitted to these 26 hospitals during the study period were included in the present analysis. Data were collected prospectively by the treating hospital physician. Information gathered for each patient included sociodemographic characteristics, comorbidities, Rankin Scale on admission, treatments during the in-hospital period, in-hospital mortality, and whether a patient was initially admitted to a stroke unit. Data collection was standardized and the documented forms were sent to the coordinating center of the Stroke Register of Northwestern Germany at the University of Muenster. Forms were scanned and checked for completeness and plausibility.

The following definitions were used: Hypertension was defined using cut-off values of measured systolic blood pressure of ≥ 140 mm Hg, diastolic blood pressure of ≥90 mm Hg, or patient self-report of treated arterial hypertension. Diabetes mellitus was defined as elevated fasting blood glucose level, a patients' self-report of a physician's diagnosis of diabetes, or use of antidiabetic drugs. Atrial fibrillation was documented by electrocardiogram or on a self-reported physician diagnosis. Previous stroke was defined as a neurological deficit of > 24 hours before the current event. Stroke was defined according to the World Health Organization criteria by the treating physician.[7] Stroke severity on admission was assessed by the treating physician using the Rankin Scale. Treatment with rtPA (intravenously and intra-arterial thrombolysis) or no thrombolysis was recorded.

### Statistical analyses

To evaluate changes in patients´ characteristics and in-hospital managements by year, we used one-way analysis of variance for continuous variables, the Cochran-Armitage test of trend for categorical variables, and the Mantel-Haenszel test of trend for ordinal variables. Mortality risk in each year was analyzed using logistic regression models (SAS Procedure GLIMMIX, statement LSMEANS), adjusted for age, sex, comorbidities, and stroke severity. Multiple imputation was used to replace missing values for comorbidities (SAS Procedure MI). Multivariable logistic regression models were applied to analyze if a change in mortality over time was mediated by different in-hospital treatment. The latter were analyzed in separate models because not all were continuously recorded. Additionally, these analyses were stratified by study time period. First, we considered only the time period in which mortality declined (from 2000 to 2008). Next, we analyzed the years 2010–2011 in which all in-hospital interventions were recorded separately. All models were adjusted for age, sex, stroke severity, sum of comorbidities, and year of admission.

Statistical significance was determined as P < 0.05. Statistical analyses were carried out using the Statistical Package of Social Sciences (SPSS) version 21 and SAS 9.4.

### Ethics

The ethics committee of the Westphalian Board of Physicians and the University of Muenster (Germany) approved the study design and waived the requirement for informed consent. Patient information was anonymized and de-identified prior to analysis.

## Results

During the 11 years, a total of 73,674 patients with ischemic stroke were registered within the 26 hospitals with 8 or more years of documentation in the Stroke Register. Of these 60 were excluded from further analysis because of an unknown year of admission. Main characteristics of the remaining 73,614 patients are given in Table 1. The number of patients admitted to the participating hospitals increased from 3,235 to 10,940 per year. During the study period, the mean age of ischemic stroke patients increased from 70.6 (13.0) to 71.9 (13.0) years. The proportion of women did not vary over time (P = 0.155). Prevalence of hypertension increased from 70.5% to 84.1% as did atrial fibrillation from 20.2% to 29.0%, and the prevalence of prior stroke from 18.9% to 26.0%, whereas the prevalence of diabetes mellitus remained roughly stable. The proportion of patients with mild and moderate stroke severity upon admission, as indicated by a Rankin Scale of 0 to 1 and 2 to 3, respectively, increased, whereas the proportion of patients with a more severe stroke, as indicated by a Rankin Scale of 4 decreased. The proportion of patients with severest stroke, as indicated by a Rankin Scale of 5 initially increased and then declined thereafter.

**Table 1:** Baseline characteristics of patients with ischemic stroke from 2000 to 2011.

	2000 (n = 3,235)	2001 (n = 4,109)	2002 (n = 4,415)	2003 (n = 3,816)	2004 (n = 4,270)	2005 (n = 4,149)	2006 (n = 4,491)	2007 (n = 7,107)	2008 (n = 7,717)	2009 (n = 9,304)	2010 (n = 10,061)	2011 (n = 10,940)	P for Trend
Demographics													
Age, mean (SD), y	70.6 (13.0)	70.5 (13.1)	70.7 (13.3)	70.6 (12.7)	70.3 (12.6)	71.0 (12.6)	71.1 (13.1)	71.5 (12.9)	71.5 (13.1)	71.7 (13.1)	71.8 (13.0)	71.9 (13.0)	< 0.001
Women, n (%)	1,618 (50.0)	2,056 (50.0)	2,170 (49.1)	1,815 (47.6)	2,007 (47.0)	1,977 (47.6)	2,141 (47.7)	3,367 (47.4)	7,717 (48.2)	9,304 (48.3)	4,851 (48.2)	5,595 (48.4)	0.155
Comorbidities, n (%)													
Hypertension	2,282 (70.5)	3,069 (74.7)	3,356 (76.0)	3,047 (79.8)	3,554 (83.2)	3,471 (83.7)	3,832 (85.3)	6,000 (84.4)	6,561 (85.0)	7,807 (84.0)	8,451 (84.0)	9,199 (84.1)	< 0.001
Diabetes mellitus	983 (30.4)	1,343 (32.7)	1,413 (32.0)	1,129 (29.6)	1,353 (31.7)	1,340 (32.3)	1,343 (29.9)	2,093 (29.4)	2,258 (29.3)	2,606 (28.0)	2,928 (29.1)	3,191 (29.2)	< 0.001
Atrial fibrillation	653 (20.2)	909 (22.1)	906 (20.5)	983 (25.8)	1,199 (28.1)	1,263 (30.4)	1,304 (29.0)	1,963 (27.6)	2,213 (28.7)	2,558 (27.5)	2,902 (28.8)	3,168 (29.0)	< 0.001
Prior stroke	612 (18.9)	746 (18.2)	744 (16.9)	952 (24.9)	1,082 (25.3)	1.072 (25,8)	1,230 (27.4)	2,047 (28.8)	2,190 (28.4)	2,563 (27.5)	2,594 (25.8)	2,847 (26.0)	< 0.001
Rankin Scale on admission, n (%)													
0	71 (2.2)	73 (1.8)	76 (1.7)	120 (3.1)	138 (3.2)	130 (3.19)	190 (4.2)	316 (4.4)	416 (5.4)	425 (4.6)	416 (4.1)	513 (4.7)	< 0.001
1	413 (12.8)	448 (10.9)	419 (9.5)	410 (10.7)	435 (10.2)	435 (10.5)	583 (13.0)	862 (12.1)	930 (12.1)	999 (10.7)	1497 (14.9)	1588 (12.8)	< 0.001
2	598 (18.5)	750 (18.3)	930 (21.1)	737 (19.3)	843 (19.7)	794 (19.1)	931 (20.7)	1,447 (20.4)	1,572 (20.4)	1,965 (21.1)	2,279 (22.7)	2,401 (22.0)	< 0.001
3	665 (20.6)	910 (22.1)	952 (20.6)	814 (21.3)	927 (21.7)	902 (21.7)	1,033 (23.0)	1,616 (22.7)	1,678 (21.7)	2,204 (23.7)	2,372 (23.6)	2,581 (20.6)	< 0.001
4	792 (24.5)	955 (23.2)	972 (22.0)	781 (20.5)	863 (20.2)	893 (21.5)	791 (17.6)	1,333 (18.8)	1,440 (18.7)	1,774 (19.1)	1,687 (16.8)	1,819 (16.6)	< 0.001
5	696 (21.5)	972 (23.7)	1,067 (24.2)	954 (25.0)	1,065 (24.9)	994 (24.0)	963 (21.4)	1,530 (21.5)	1,682 (21.8)	1,937 (20.8)	1,811 (18.0)	2,037 (18.6)	< 0.001

**Fig 1:**
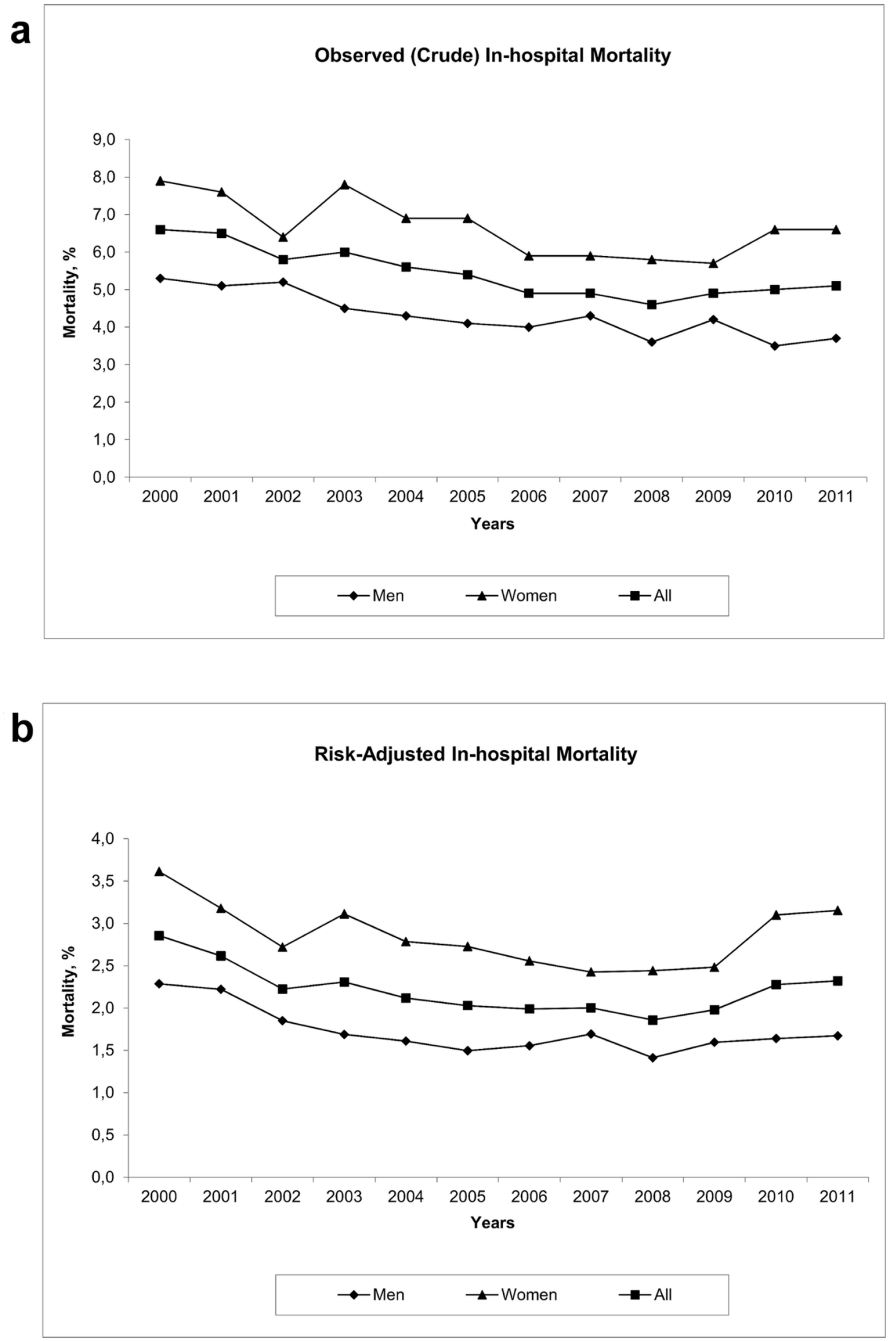
In-hospital mortality among patients with ischemic stroke between 2000 and 2011. A, Observed (crude) mortality. B, Risk-adjusted mortality was determined with the use of logistic regression models to adjust for age, sex, initial stroke severity, and the number of comorbidities.

The observed, crude in-hospital mortality decreased from 6.6% in 2000 to 4.6% in 2008 (P < 0.001 for trend) and then remained fairly stable (Fig 1a). The crude mortality of women declined from 7.9% in 2000 to 5.7% in 2009 and then slightly increased thereafter to 6.6% in 2011. The crude mortality of men declined from 5.3% in 2000 to 3.6% in 2008 and then remained stable. After adjustment for age, sex, stroke severity upon admission, and comorbidities, the overall mortality decreased gradually from 2.85% in 2000 to 1.86% in 2008 (P < 0.001 for trend) and then slightly increased to 2.32% in 2011 (Fig 1b). The adjusted mortality of women declined from 3.61% to 2.43% in 2008 and increased to 3.15% in 2011 and the adjusted mortality of men declined from 2.29% to 1.41% in 2008 and then slightly increased to 1.67% in 2011.

The use of intravenous and intra-arterial thrombolysis increased over time, from 2.9% to 15.4% (Table 2). During the study period use of antiplatelet therapy within the first 48 hours from admission increased from 54.0% to 81.5%. Use of antihypertensive therapy, antidiabetics, and statins were recorded only after 2002. Antihypertensive therapy and statin use markedly increased from 73.1% to 84.4% and 32.0% to 68.0%, respectively, whereas antidiabetic use only slightly increased from 22.1% to 26.0%. Use of anticoagulants gradually increased from 12.9% to 27.6% as did physiotherapy use from 73.8% to 88.4%. The proportion of patients who were admitted to a stroke unit increased during the study period from 57.1% to 85.3%.

**Table 2:** In-hospital management of patients with stroke from 2000 to 2011.

	2000 (n = 3,235)	2001 (n = 4,109)	2002 (n = 4,415)	2003 (n = 3,816)	2004 (n = 4,270)	2005 (n = 4,149)	200 (n = 4,491)	2007 (n = 7,107)	2008 (n = 7,717)	2009 (n = 9,304)	2010 (n = 10,061)	2011 (n = 10,940)	P for Trend
Thrombolysis‡, n (%)	94 (2.9)	121 (2.9)	178 (4.0)	162 (4.2)	263 (6.2)	309 (7.4)	356 (8.4)	581 (10.1)	762 (11.9)	1172 (13.5)	1486 (14.8)	1689 (15.4)	< 0.001
Other in-hospital treatments, n (%)													
Antiplatelet therapy within 48 hours	1,489 (54.0)	2,291 (55.8)	2,688 (60.9)					5679 (84.0)	6351 (85.3)	7519 (82.8)	8255 (82.9)	8848 (81.5)	< 0.001
Antihypertensive therapy				2782 (73.1)	3333 (78.1)	3304 (79.6)	3362 (83.0)	4490 (85.0)	4618 (85.7)	6123 (84.2)	8241 (83.4)	9126 (84.4)	< 0.001
Antidiabetics				843 (22.1)	1062 (24.9)	1045 (25.2)	970 (24.3)	1259 (24.2)	1289 (24.5)	1682 (23.4)	2554 (25.4)	2801 (26.0)	< 0.001
Statins				1217 (32.0)	1577 (36.9)	1706 (41.1)	1936 (48.5)	3135 (61.6)	3597 (69.0)	4651 (65.1)	6443 (65.7)	7293 (68.0)	< 0.001
Anticoagulants	417 (12.9)	568 (13.8)	581 (13.2)	529 (13.9)	593 (13.9)	653 (15.7)	819 (18.7)	1381 (20.9)	1514 (20.6)	2078 (23.1)	2369 (24.0)	2990 (27.6)	< 0.001
Physiotherapy	2388 (73.8)	3311 (80.6)	3534 (80.0)	2940 (77.0)	3316 (77.7)	3281 (79.1)	3661 (82.8)	5960 (87.9)	6741 (89.8)	8431 (92.2)	8763 (88.2)	9661 (88.4)	< 0.001
Admission to Stroke Unit, n (%)	1552 (57.1)	2341 (62.2)	2647 (63.7)	2139 (57.1)	2279 (54.4)	2486 (61.2)	1533 (68.9)				8400 (84.9)	9245 (85.3)	< 0.001

^‡^ Includes intravenous and intra-arterial thrombolysis.

Percentages are related to available informations and therefore sum to 100%. Blank cells indicate data not available. Data missing rate: for admission ward 7.9%.

In multivariable logistic regression analyses we adjusted for age, sex, stroke severity, sum of comorbidities, year of admission, and we separately adjusted for different in-hospital managements, since these were not continuously recorded (Table 3). We thus evaluated whether different treatments mediated the decline in case fatality. The in-hospital mortality significantly decreased per year with adjusted OR of 0.95 and 0.96 (maximum 95% CIs, 0.92 to 0.99) in different logistic regression models (Table 3). The association between in-hospital mortality and year of admission was significantly modified by treatment with antiplatelets within 48 hours and by physiotherapy (Table 3). By adding these therapies the effect of the year of admission on mortality was attenuated from an OR of 0.96 (95% CI, 0.94–0.98) to 0.99 (0.97–1.01) and from an OR of 0.96 (95% CI, 0.94–0.97) to 0.99 (0.97–1.00), respectively. The associations of the year of admission with mortality became insignificant when taking antiplatelet therapy within 48 hours and physiotherapy into account (P = 0.302 and P = 0.139). The association between different in-hospital treatments and mortality are shown in S1 Table. Thrombolysis was independently associated with an increase in the odds of dying in the hospital, whereas all other treatments and admission to a stroke unit were significantly associated with a decrease in the odds of dying in the hospital. In a multivariable logistic regression analysis for the years 2010 and 2011, in which all treatments were recorded, all treatments except thrombolysis and antidiabetics were significantly associated with decreased in-hospital mortality (S1 Table).

**Table 3:** Logistic regression analyses* showing effects of in-hospital managements on the annual decrease in in-hospital mortality.

	aOR (95% CI)	P
**Model 1, effect of thrombolysis** ‡		
Decline in mortality per year, not adjusted for thrombolysis	0.96 (0.94–0.97)	< 0.001
Decline in mortality per year, adjusted for thrombolysis	0.94 (0.93–0.96)	< 0.001
**Model 2, effect of antiplatelet therapy within 48 hours**		
Decline in mortality per year, not adjusted for antiplatelets	0.96 (0.94–0.98)	< 0.001
Per year, adjusted including antiplatelets	0.99 (0.97–1.01)	0.302
**Model 3, effect of antihypertensive therapy**		
Decline in mortality per year, not adjusted for antihypertensives	0.96 (0.93–0.99)	0.011
Per year, adjusted including antihypertensives	0.94 (0.90–0.97)	< 0.001
**Model 4, effect of antidiabetics**		
Decline in mortality per year, not adjusted for antidiabetics	0.96 (0.93–0.99)	0.011
Per year, adjusted* including antidiabetics	0.89 (0.86–0.92)	< 0.001
**Model 5, effect of statins**		
Decline in mortality per year, not adjusted for statins	0.96 (0.93–0.99)	0.011
Per year, adjusted* including statins	0.95 (0.92–0.99)	0.013
**Model 6, effect of anticoagulants**		
Decline in mortality per year, not adjusted for anticoagulants	0.96 (0.94–0.97)	< 0.001
Per year, adjusted* including anticoagulants	0.96 (0.94–0.97)	< 0.001
**Model 7, effect of physiotherapy**		
Decline in mortality per year, not adjusted for physiotherapy	0.96 (0.94–0.97)	< 0.001
Per year, adjusted* including physiotherapy	0.99 (0.97–1.00)	0.139
**Model 8, effect of admission to Stroke Unit**		
Decline in mortality per year, not adjusted for stroke unit admission	0.95 (0.92–0.97)	< 0.001
Per year, adjusted* including stroke unit admission	0.94 (0.91–0.97)	< 0.001

* All models were adjusted for age, sex, stroke severity on admission indicated by Rankin Scale, sum of comorbidities, year of admission, and separately for different in-hospital managements.

^‡^ Includes intravenous and intra-arterial thrombolysis. Models including thrombolysis considered the time period 2000–2008, models including antiplatelets considered the time period 2000–2002 and 2007–2008, models including antihypertensive therapy, antidiabetic, and statins considered the time period 2003–2008, models including anticoagulants and physiotherapy considered the time period 2000–2008, modes including admission to Stroke Unit considered the time period 2000–2006.

Abbreviations: aOR, adjusted odds ratio; CI, confidence interval

## Discussion

Using the data base of a large German stroke register we observed a decline of in-hospital mortality of patients with ischemic stroke of absolute 2% and relatively 33% between the years 2000 and 2008. The reduction in mortality was accompanied by changes in patient management, such as more frequent use of thrombolysis, antiplatelet therapy within 48 hours, anticoagulants, physiotherapy, and statins. Patient characteristics also changed during the study period considerably. The proportion of patients with mild and moderate stroke severity upon admission increased, while the proportion of those with more severe stroke decreased. Over time the average number of comorbidities per patient increased. The significant effect of time on the decreasing mortality was substantially attenuated by antiplatelet therapy within 48 hours and physiotherapy, suggesting that these two interventions partly mediated the decrease in mortality over time.

Previous studies also reported a decreased short-term mortality after ischemic stroke in a time period comparable to that in our study.[3,8,9] This decline could not be attributed to changes in stroke incidence alone but to a decreased case fatality. If the implementation of evidence-based therapies in routine care contributed to the declining mortality has not been investigated so far. Patient related factors, such as older age, higher stroke severity, female sex, and the comorbidity burden were independent predictors of short-term mortality in prior studies.[8,10] However, even after adjustment for these characteristics the decline in case fatality remained significant in our study, indicating additional explaining factors. One previous observational study showed that the in-hospital mortality decreased between 2004 and 2010 after the use of the clopidogrel had increased.[11] However, the association between these descriptive observations was not analyzed in more detail. There is evidence from a meta-analysis of randomized clinical trials that aspirin, when started within 48 hours after stroke reduces mortality.[12] In contrast, the effect of physiotherapy on stroke mortality is less clear.[13] Although not convincingly demonstrated for physiotherapy itself, it was shown that mobilization after stroke reduces the likelihood of complications, such as infections and deep vein thrombosis,[4] which in turn might contribute to a decrease in mortality. In our study significantly more deaths occurred in patients treated with rtPA in the years 2000 to 2008, whereas mortality after thrombolysis was not increased in the model considering the years 2010 and 2011. Thrombolysis is well established to improve the neurological outcome after stroke. However, our inconclusive results on the in-hospital mortality after thrombolysis are in accordance with previous observations. The IST-3 (the third international stroke trial) study, in which patients received thrombolysis beyond the established therapeutic time window of 4.5 hours mortality was increased in the treatment group compared to placebo. In contrast, The European Cooperative Acute Stroke Study (ECASS) III reported no excess of deaths after rtPA treatment.[14] Commonly, it is assumed that the increasing use of thrombolysis cannot account for reductions in ischemic stroke mortality.[15]

Our study has strengths and limitations. Among the strengths is that data were collected in a uniform, prospective way over a long time period in 26 hospitals. Patient characteristics known to be associated with early mortality after ischemic stroke, such as age, comorbidity burden, and the initial stroke severity were included in the statistical models. However, our findings should be interpreted in light of the following potential limitations. As in any observational study a definitive causal relationship between the effect of changes of treatments and mortality cannot be made. Although we collected key variables over time and found them to mediate the decrease in mortality, the possibility remains, that other external factors also changed over time and thereby confound the results. In addition, diagnostic procedures and accuracy might have improved over time. In particular the increased prevalence of atrial fibrillation might be due to higher detection rates and thus be overrated. Further limitations are missing information on some in-hospital treatments during the study period and on the National Institutes of Health Stroke Scale (NIHSS) on admission. Moreover, we used strict inclusion criteria and included only those hospitals that documented patients during a substantial part of the study period. Therefore the generalizability of our findings might be limited. Finally, patients in our study were observed only during the period of hospitalization. No information on post-discharge outcomes is currently collected in the present register so time trends on long-term mortality are not available.

## Conclusion

In a large German stroke register in-hospital mortality of ischemic stroke patients decreased by about one third between 2000 and 2008. The decline in mortality was accompanied by overall improvements in patient management and, in particular, mediated by the application of early antiplatelet therapy and physiotherapy.

## Supporting Information

S1 Appendix (DOC)

S1 Checklist (DOC)

S1 Table (DOC)

## References

[1] Lozano R, Naghavi M, Foreman K, Lim S, Shibuya K, Aboyans V, et al (2012) Global and regional mortality from 235 causes of death for 20 age groups in 1990 and 2010: a systematic analysis for the Global Burden of Disease Study 2010. Lancet 380: 2095–2128. 10.1016/S0140-6736(12)61728-0 23245604 PMC10790329

[2] Carandang R, Seshadri S, Beiser A, Kelly-Hayes M, Kase CS, Kannel WB, et al (2006) Trends in incidence, lifetime risk, severity, and 30-day mortality of stroke over the past 50 years. JAMA 296: 2939–2946.. 17190894 10.1001/jama.296.24.2939

[3] Vaartjes I, O’Flaherty M, Capewell S, Kappelle J, Bots M (2013) Remarkable Decline in Ischemic Stroke Mortality is Not Matched by Changes in Incidence. Stroke 44: 591–597. 10.1161/STROKEAHA.112.677724 23212165

[4] Jauch EC, Saver JL, Adams HP Jr, Bruno A, Connors JJB, Demaerschalk BM, et al (2013) Guidelines for the early management of patients with acute ischemic stroke: a guideline for healthcare professionals from the american heart association/american stroke association. Stroke 44: 870–947. 10.1161/STR.0b013e318284056a 23370205

[5] Teuschl Y, Brainin M, Matz K, Dachenhausen A, Ferrari J, Seyfang L, et al (2013) Time trends in patient characteristics treated on acute stroke-units: results from the austrian stroke unit registry 2003–2011. Stroke 44: 1070–1074. 10.1161/STROKEAHA.111.676114 23412371

[6] Minnerup J, Wersching H, Ringelstein EB, Schilling M, Schäbitz W-R, Wellmann J, et al (2011) Impact of the extended thrombolysis time window on the proportion of recombinant tissue-type plasminogen activator-treated stroke patients and on door-to-needle time. Stroke 42: 2838–2843. 10.1161/STROKEAHA.111.616565 21852612

[7] Hatano S (1976) Experience from a multicentre stroke register: a preliminary report. Bull World Health Organ 54: 541–553. 1088404 PMC2366492

[8] Schmidt M, Jacobsen JB, Johnsen SP, Bøtker HE, Sørensen HT (2014) Eighteen-year trends in stroke mortality and the prognostic influence of comorbidity. Neurology 82: 340–350. 10.1212/WNL.0000000000000062 24363134

[9] Towfighi A, Tai W, Markovic D, Ovbiagele B (2011) Sex-specific temporal trends in in-hospital mortality after stroke among middle-age individuals in the United States. Stroke 42: 2740–2745. 10.1161/STROKEAHA.110.612648 21799158

[10] Ovbiagele B (2010) Nationwide trends in in-hospital mortality among patients with stroke. Stroke 41: 1748–1754. 10.1161/STROKEAHA.110.585455 20558829

[11] Tanne D, Koton S, Molshazki N, Goldbourt U, Shohat T, Tsabari R, et al (2012) Trends in management and outcome of hospitalized patients with acute stroke and transient ischemic attack: the National Acute Stroke Israeli (NASIS) registry. Stroke 43: 2136–2141. 10.1161/STROKEAHA.111.647610 22569935

[12] Sandercock PAG, Counsell C, Gubitz GJ, Tseng M-C (2008) Antiplatelet therapy for acute ischaemic stroke. Cochrane Database Syst Rev: CD000029 10.1002/14651858.CD000029.pub2 18646056

[13] Saunders DH, Sanderson M, Brazzelli M, Greig CA, Mead GE (2013) Physical fitness training for stroke patients. Cochrane Database Syst Rev 10. 10.1002/14651858.CD003316.pub5 24142492

[14] Hacke W, Kaste M, Bluhmki E, Brozman M, Dávalos A, Guidetti D, et al (2008) Thrombolysis with alteplase 3 to 4.5 hours after acute ischemic stroke. N Engl J Med 359: 1317–1329. 10.1056/NEJMoa0804656 18815396

[15] Lackland DT, Roccella EJ, Deutsch AF, Fornage M, George MG, Howard G, et al (2014) Factors influencing the decline in stroke mortality: a statement from the American Heart Association/American Stroke Association. Stroke 45: 315–353. 10.1161/01.str.0000437068.30550.cf 24309587 PMC5995123

