# Supplementary material for: Explaining the Decrease of In-Hospital Mortality from Ischemic Stroke
Source: PLoS One. 2015 Jul 8;10(7):e0131473. doi: 10.1371/journal.pone.0131473 (PMC4496086; doi:10.1371/journal.pone.0131473)
Supplement: S1 Appendix — (DOC) [file pone.0131473.s001.doc]

**Appendix**

**Supplemental Methods**

Participating hospitals within the stroke register ´Northwest Germany´ document each admitted stroke patient on a standardized form, either electronically or on paper.

### Stroke Register of Northwestern Germany

For the present study we used data from the following hospitals: Johannes Wesling Klinikum Minden; Krankenhaus Lübbecke; St. Josef-Hospital Bochum; BG Universitätsklinikum Bergmannsheil GmbH Bochum; Gemeinschaftskrankenhaus Herdecke; Evangelisches Krankenhaus GmbH Gelsenkirchen; Universität Münster; St. Marien-Hospital Hamm; St. Barabara Klinik Heesen; St. Johannes Hospital Hagen; Städtisches Krankenhaus Arnsberg; Klinikum Lippe-Lemgo; St. Vincenz Krankenhaus Landeshospital Paderborn; Städtische Kliniken Dortmund; Knappschaftskrankenhaus Dortmund; Hüttenhospital Dortmund; Kath. Krankenhaus Dortmund-West; Knappschaftskrankenhaus Recklinghausen; Christophorus-Kliniken GmbH Dülmen; Evangelisches Krankenhaus Castrop-Rauxel; Elisabeth-Krankenhaus Recklinghausen; Knappschaftskrankenhaus Bottrop; Klinikum Osnabrück; Hans-Susemihl-Krankenhaus Emden; Ev. Bathildiskrankenhaus Bad Pyrmont gGmbH; Helios Klinikum Wuppertal-Barmen.
