# Supplementary figures and images for: Explaining the Decrease of In-Hospital Mortality from Ischemic Stroke
Source: PLoS One. 2015 Jul 8;10(7):e0131473. doi: 10.1371/journal.pone.0131473 (PMC4496086; doi:10.1371/journal.pone.0131473)

**
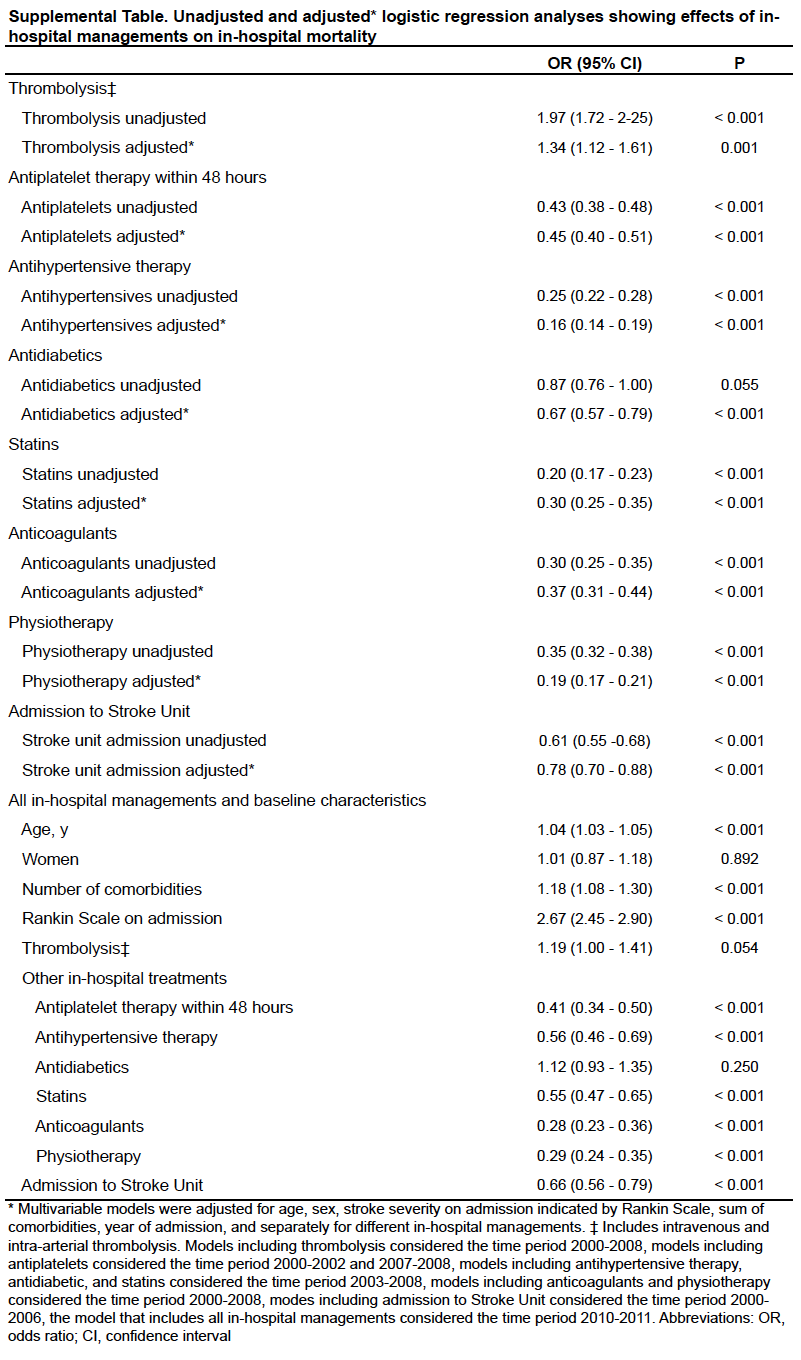
**

Supplement: S1 Table — (DOC) [file pone.0131473.s003.doc]
